# Potential Schizophrenia Disease-Related Genes Prediction Using Metagraph Representations Based on a Protein-Protein Interaction Keyword Network: Framework Development and Validation

**DOI:** 10.2196/50998

**Published:** 2023-11-15

**Authors:** Shirui Yu, Ziyang Wang, Jiale Nan, Aihua Li, Xuemei Yang, Xiaoli Tang

**Affiliations:** 1 Institute of Medical Information Chinese Academy of Medical Sciences Beijing China

**Keywords:** disease gene prediction, metagraph, protein representations, schizophrenia, keyword network

## Abstract

**Background:**

Schizophrenia is a serious mental disease. With increased research funding for this disease, schizophrenia has become one of the key areas of focus in the medical field. Searching for associations between diseases and genes is an effective approach to study complex diseases, which may enhance research on schizophrenia pathology and lead to the identification of new treatment targets.

**Objective:**

The aim of this study was to identify potential schizophrenia risk genes by employing machine learning methods to extract topological characteristics of proteins and their functional roles in a protein-protein interaction (PPI)-keywords (PPIK) network and understand the complex disease–causing property. Consequently, a PPIK-based metagraph representation approach is proposed.

**Methods:**

To enrich the PPI network, we integrated keywords describing protein properties and constructed a PPIK network. We extracted features that describe the topology of this network through metagraphs. We further transformed these metagraphs into vectors and represented proteins with a series of vectors. We then trained and optimized our model using random forest (RF), extreme gradient boosting, light gradient boosting machine, and logistic regression models.

**Results:**

Comprehensive experiments demonstrated the good performance of our proposed method with an area under the receiver operating characteristic curve (AUC) value between 0.72 and 0.76. Our model also outperformed baseline methods for overall disease protein prediction, including the random walk with restart, average commute time, and Katz models. Compared with the PPI network constructed from the baseline models, complementation of keywords in the PPIK network improved the performance (AUC) by 0.08 on average, and the metagraph-based method improved the AUC by 0.30 on average compared with that of the baseline methods. According to the comprehensive performance of the four models, RF was selected as the best model for disease protein prediction, with precision, recall, F1-score, and AUC values of 0.76, 0.73, 0.72, and 0.76, respectively. We transformed these proteins to their encoding gene IDs and identified the top 20 genes as the most probable schizophrenia-risk genes, including the EYA3, CNTN4, HSPA8, LRRK2, and AFP genes. We further validated these outcomes against metagraph features and evidence from the literature, performed a features analysis, and exploited evidence from the literature to interpret the correlation between the predicted genes and diseases.

**Conclusions:**

The metagraph representation based on the PPIK network framework was found to be effective for potential schizophrenia risk genes identification. The results are quite reliable as evidence can be found in the literature to support our prediction. Our approach can provide more biological insights into the pathogenesis of schizophrenia.

## Introduction

### Background

Schizophrenia is a serious mental disease characterized by abnormalities in thinking and cognition [1], whose occurrence is widely believed to be closely related to genetics and gene expression. This chronic and disability-causing disease not only has great effects on the quality of life of patients but also imposes a heavy burden on their families and society as a whole; therefore, schizophrenia has long been a key focus of research in the medical field [2]. Searching for the associations between diseases and genes is an effective way to study complex diseases, allowing an in-depth exploration of the mechanisms and molecular basis of diseases, which is crucial to establishing accurate treatments and diagnoses.

Numerous network-based approaches have recently been proposed for disease-gene association prediction, such as protein-protein interaction (PPI), gene regulatory, gene coexpression, and metabolic interaction networks. Among these molecular networks, PPI networks are widely used as a conducive approach to discover potential disease-causing genes [3] since proteins work together to perform common biological functions. In addition, proteins associated with a common set of biological properties tend to have common topological properties in the network, such as node degree and centrality, and the pathways elucidating disease mechanisms are typically represented as strongly connected paths in the PPI network [4].

However, existing PPI networks are often incomplete and noisy. Thus, it is necessary to collect multiple types of data and build an integrated network that includes multiple, heterogeneous types of resources. This method will greatly extend the scope and ability for disease gene prediction [5]. However, scientific data do not consider the properties of biological data themselves, whereas the scientific literature represents a record of the latest scientific discoveries and important research results, containing a large amount of additional biological knowledge such as protein biological functions and sequence characteristics. Therefore, data from the scientific literature along with primary scientific data can be used as a complement to discover the implicit information and enhance the ability to predict disease-associated genes.

Many new techniques have been developed to study heterogeneous networks containing multimodal biological data. Recently, a general framework that considers heterogeneity by defining type-specific graphs was introduced [6]. The advantage of metagraph-based approaches is that they capture rich semantics, comprehensively represent different features, and effectively identify influential components of the given network, making it possible to preserve the network structure and providing flexibility to explore a diverse set of descriptors.

The problem of disease-related genes prediction tackled in our study can be regarded as a disease protein classification problem that aims to identify disease-related proteins, and the proteins are then linked to their gene products to obtain disease-related genes. The goal is to train a classifier to judge whether the protein is associated with a disease based on a training set, and we can then predict each protein’s likelihood of falling into a given category in the test set with minimal prediction errors using the learned protein features.

In this study, we attempted to integrate the PPI network from the STRING database with the keywords in the UniProt database to form a heterogeneous PPI-keyword (PPIK) network for disease protein prediction. Based on the PPIK network, we extended the metagraph methodology to predict the probability that an association between a gene and disease exists. Each protein was represented as a series of metagraphs. A previous study that utilized metagraph representations to exploit a keywords-supplemented PPI network achieved good performance on predictions for breast cancer [7]. We made some improvement on the basis of this method. In particular, we extracted basic metagraph structures from the network to represent proteins, which fully revealed the traits of proteins. We further considered the use of a more appropriate algorithm for metagraph representations that is suitable for complex networks to capture more implicit features. In addition, we used the latest machine learning models to understand the ever-growing scientific data, which were applied to schizophrenia as a more specific disease to enable better disease-gene association prediction, thereby uncovering potential therapeutic targets.

### Related Work

#### Pathogenesis Analysis

In recent decades, massive efforts have been made to identify the mechanisms underlying pathogenesis and explore the genetic associations of disease. Genome-wide association studies (GWAS) have been established as a main strategy to solve this problem. This method infers genome intervals that are involved in genetic diseases. However, GWAS is a time-consuming and expensive task. To resolve such issues, several other strategies have been proposed in recent years. Gene-set enrichment analysis identifies prevalent biological functions among genes contained in disease-associated loci. For example, Segrè et al [8] developed the meta-analysis gene-set enrichment of variant associations (MAGENTA) method to test whether sets of functionally related genes are enriched for associations with a polygenic disease or trait. Network-based approaches exploit topological characteristics of the network, which is instrumental in understanding the interactions and pathways in the context of diseases. For example, Liekens et al [9] used data from 21 publicly available curated databases and built a network called BioGraph to identify relations between heterogeneous biomedical entities. Literature mining techniques aim to chronicle the relatedness of genes to identify a subset of highly related associated genes. For example, Raychaudhuri et al [10] reported the Gene Relationships Among Implicated Loci (GRAIL) algorithm as an approach to assess relationships among genomic disease regions by text mining of PubMed abstracts.

#### Network-based Approach

The network-based approach links diverse aspects of diseases in a whole-system view, further reveals the useful knowledge implied in the network using powerful computational and logical reasoning abilities, and tracks potential disruptions on biological pathways due to the disease-causing factors. This approach is conducive for disease biology analysis [11]. The network-based approaches for disease-causing genes prediction generally fall into three categories, including methods using graph-theoretic algorithms, those using machine learning algorithms, and those using graph representation learning methods [12]. In the graph-theoretic methods, the simplest approach is direct neighbor counting, which involves checking whether two genes are connected directly in a molecular network. Module-based methods hypothesize that proteins within the same topological or functional module on a network are more likely to be associated with the same disease. Diffusion-based methods are proposed to predict the gene-disease relation using the global network structure [13]. These methods anchor upon known disease proteins as seeds, which propagate along the network through random walks. The machine learning–based methods use traditional machine learning models, positive and unlabeled learning, or deep learning to predict disease-gene associations [14]. For example, a method called BRIDGE [15] was introduced for prioritization of disease genes by integrating various gene aspects through a weighting scheme. This scheme was attained through a multiple linear regression model with a least absolute shrinkage and selection operator penalty, which determined the phenotypic similarity between two diseases based on the functional similarities between their associating genes. In the graph representation learning methods, the latent features for the nodes are automatically learned. For example, matrix factorization techniques are useful for revealing important associations between diseases and genes. The GeneHound method was proposed based on Bayesian probabilistic matrix factorization for addressing the disease gene prioritization problem, which jointly learns the gene and disease latent factors and constructs corresponding gene- and disease-association matrices to predict disease-gene associations [16]. Graph embedding is another network-based method, which learns the low-dimensional and continuous vector representations of nodes through a neural network. For example, the SkipGram architecture is an extensively used architecture to construct associations between the node and its neighborhood [17].

Recently, with development in the area of machine learning and deep learning, deep learning–based techniques have shown bright prospects for handling network data. Several methods have been proposed in the field of disease-causing genes prediction, as well as some similar fields such as drug-disease association prediction. For example, a method that combines two graph convolutional networks (GCNs) and matrix factorization was proposed to predict gene-disease associations. In this model, diseases, gene features, and similarity graphs are input to two parallel GCNs, which combine their obtained embeddings through an inner product to obtain the prediction, demonstrating effectiveness in capturing useful information from the network [18]. The relations-enhanced drug-disease association prediction method was assembled with three attention mechanisms, which can sequentially learn drug/disease representations by a general heterogeneous GCN-based node embedding block, topological subnet embedding block, graph attention block, and layer attention block. This model enhanced the performance of drug-disease association prediction [19].

## Methods

### Design

**Figure 1:**
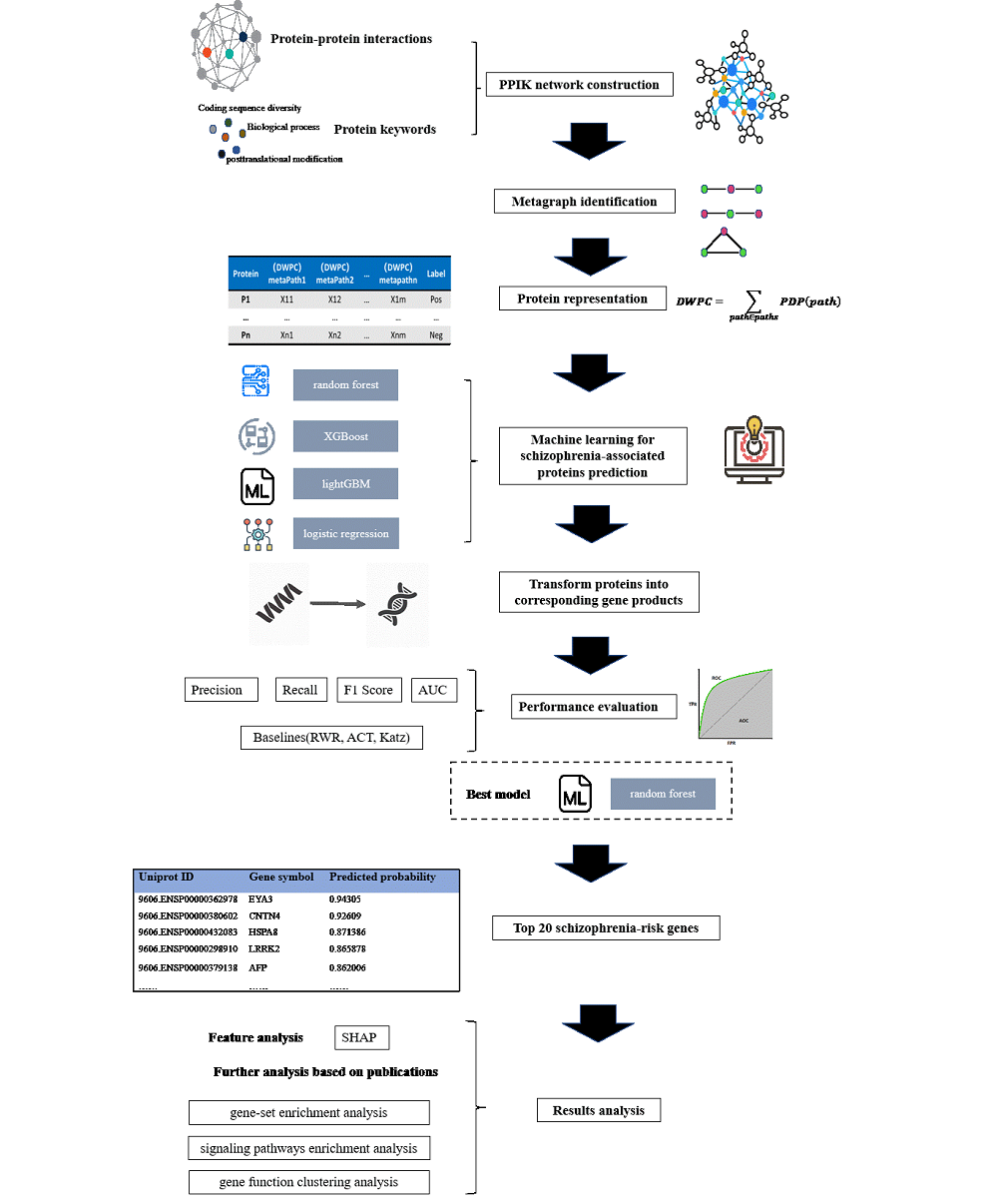
General framework of the proposed method. ACT: average commute time; AUC: area under the receiver operating characteristic curve; DWPC: degree-weighted path count; ML: machine learning; PDP: path-degree product; PPIK: protein-protein interaction-keywords; RWR: random walk with restart; SHAP: Shapley additive explanation.

We employed metagraph representations based on the PPIK network method to improve disease-associated genes prediction. The general framework is shown in Figure 1.

### PPIK Network Construction

A PPI network is frequently noisy and incomplete. The keywords in the UniProt database represent annotated information based on the literature or empirical evidence, covering various biological aspects of proteins [20], as summarized in Table 1. As disease-associated proteins are likely to share similar properties, these properties may reveal their function. Furthermore, proteins with the same domain as the identified disease proteins could also be associated with the disease. Therefore, we developed a method to associate these properties with different proteins together to complement existing PPI networks, which could encode both the interactive functions and biological properties of proteins. We used data in the UniProt database to directly construct the relations between proteins and keywords. Since each protein corresponds to multiple keywords in the database, we established the links between proteins and keywords based on this correspondence. On the one hand, protein-keyword associations can reinforce useful PPIs. On the other hand, proteins with no direct interactions can newly become related through keywords.

**Table 1:** Summary of keywords from the UniProt database.

Keyword category	Examples
Biological process	Apoptosis, cell cycle, cAMP biosynthesis
Cellular component	Golgi apparatus, vacuole, cytoplasm
Coding sequence diversity	Polymorphisms, RNA-editing, alternative splicing
Domain	SH2 domain, Kelch repeat, transmembrane
Ligand	cAMP, S-adenosyl-l-methionine, cGMP
Molecular function	RNA-binding, protein kinase inhibitor
Posttranslational modification	Phosphorylation, ubiquitination, acetylation
Technical term	Allosteric enzyme, transposable element

**Figure 2:**
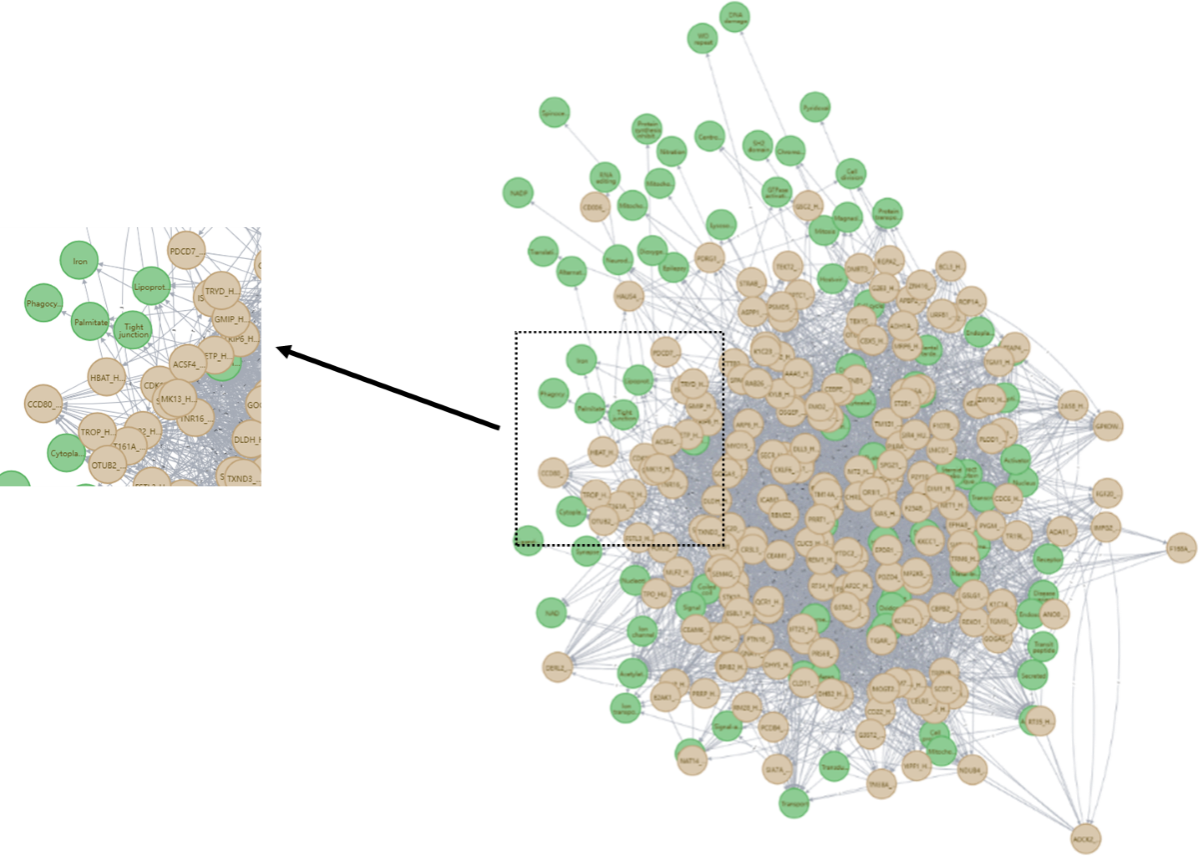
Part of the protein-protein interaction-keyword (PPIK) network based on the UniProt and STRING databases. In this network, the yellow nodes represent proteins and the green nodes represent keywords. A zoomed-in view of part of the PPIK network is shown on the left. Here, we can see that nodes with different protein and keyword labels connect with each other.

In this study, we focused only on human proteins. We obtained PPIs from the STRING database and exploited protein keywords from the UniProt database to construct the PPIK network. The network contains two types of nodes, including protein and keyword nodes. The relationships in the network indicate the interaction between two proteins and the association between the protein and keyword, which are not distinguished. A representative PPIK network is shown in Figure 2.

### Metagraph Identification

**Figure 3:**
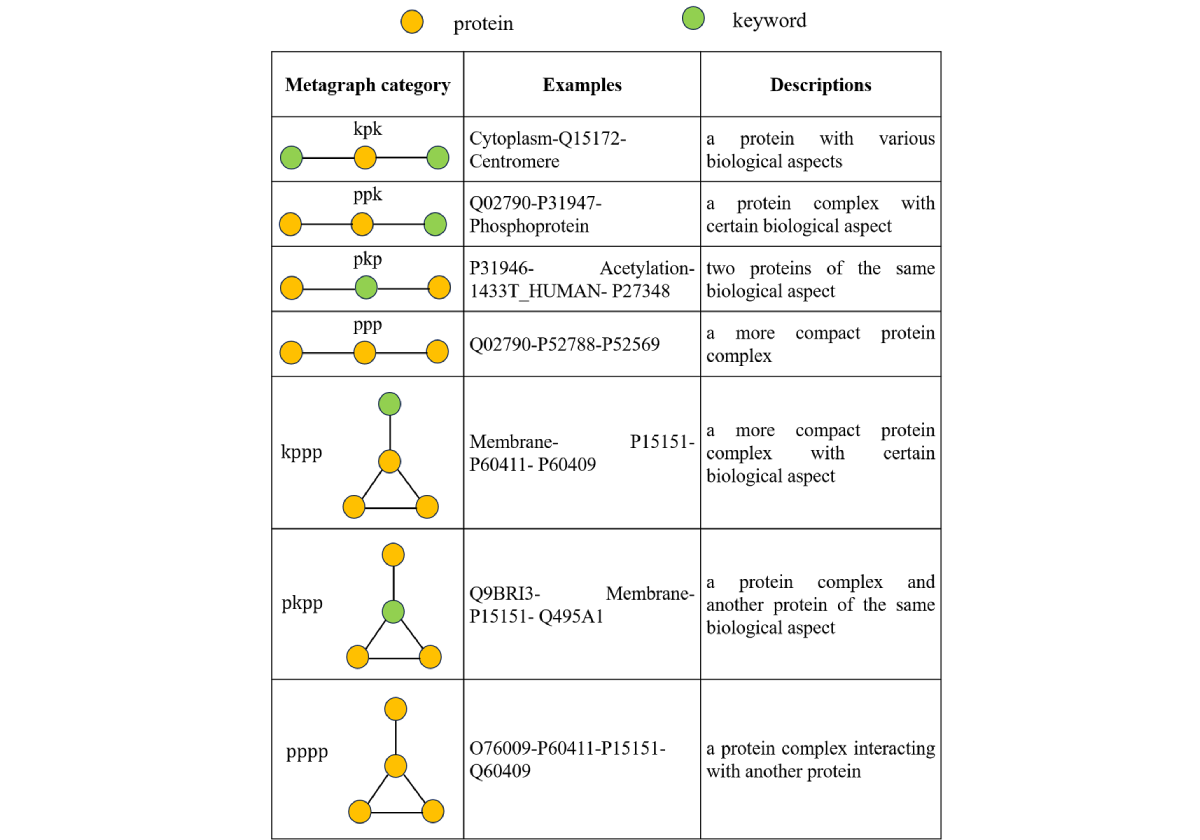
Seven types of metagraphs extracted from the protein-protein interaction-keywords (PPIK) network. The left column shows the structures of the seven metagraphs on the PPIK network and the middle and right columns provide examples and descriptions of each type of metagraph, respectively.

A metagraph is a graph structure capturing a particular topology of the network, which can obtain the location information of each node and the type-specific path pattern between a source node and a destination node in the network. Leveraging a metagraph to characterize a network will not only preserve the structural features but will also help to identify rich pathway information, which offers strong flexibility in heterogeneous networks [21]. A heterogeneous PPIK network comprises both proteins and keywords. Proteins with similar functional roles such as their disease-causing property tend to interact with other proteins and associate with certain keywords in a similar arrangement on the PPIK network. Thus, proteins with similar roles tend to have similar topological characteristics. To model topological similarities, we used a metagraph approach to define the specific structure and differentiated between different labels of nodes on the PPIK network. Each metagraph describes a particular heteronomous biological arrangement between one or more proteins and keywords. Two proteins associated with the same metagraph tend to have similar functional roles. Therefore, we could use different types of metagraphs to represent each protein, while identifying its interactions with other proteins and associations with keywords. Each instance of a metagraph represents specific evidence of the proteins’ functional properties. In particular, we only considered metagraphs of three and four nodes, which represented a good balance between efficiency and accuracy. We defined seven types of metagraphs to reveal the network feature and their common structures are shown in Figure 3. The seven metagraphs cover all instances of network motifs with three and four nodes on the PPIK network, and thus enable the thorough capture of structural characteristics of the heterogeneous network, which in turn allows for an accurate representation of protein features.

### Proteins Representation

We used a series of metagraphs to construct the vector representation of each protein to characterize its multiple features. To compute the prevalence of a type-specific metagraph, we employed a type-specific metapath counts approach known as the degree-weighted path count (DWPC) [22], which represents an improvement of the path count metric, as a basic social network analysis method, by dampening each edge between a source and target node as an effective feature extraction methodology accommodating a heterogeneous network of any size. This method involves first calculating the path-degree product (PDP) of each edge. All metaedge-specific degrees along the path (*D_path_*) are determined, where each edge composing the path contributes two degrees. Subsequently, each degree is raised to the *–w* power, where *w*≥0, which is known as the damping exponent. This parameter adjusts the intensity for each path traversing highly connected nodes to be down-weighted, and the best performance is usually achieved with *w*=0.4. Finally, all exponentiated degrees are multiplied to yield the PDP of each path according to the following formula:

*PDP(path)*=Π*_d∈Dpath_* d*^–w^*
**(1)**

The DWPC is then calculated as the sum of PDPs:

DWPC=∑*_path∈paths_PDP(path)*
**(2)**

**Figure 4:**
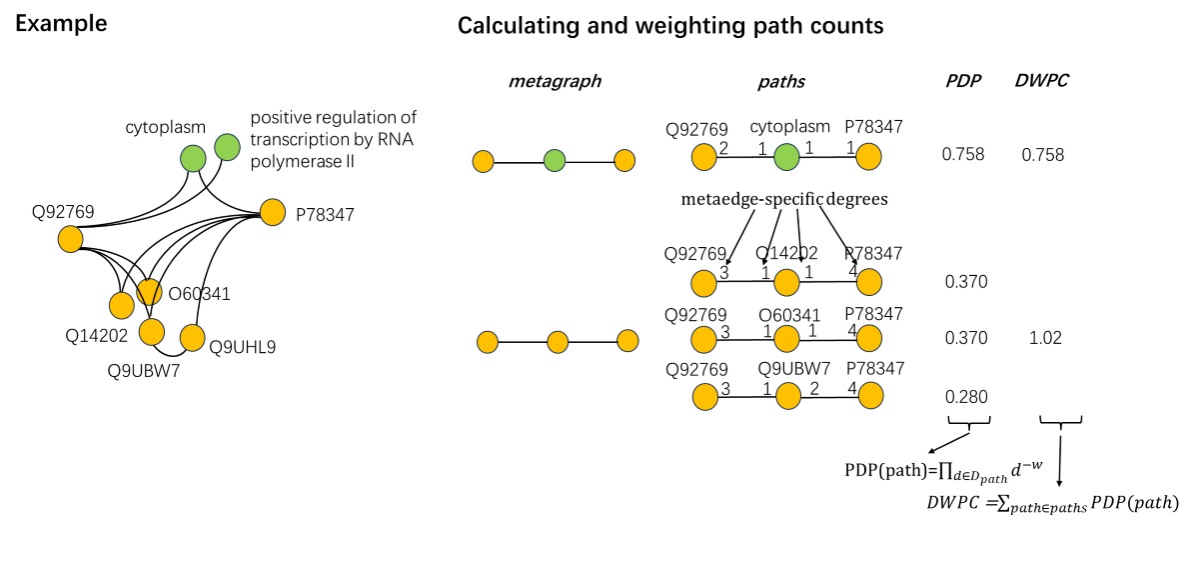
Computation process for the degree-weighted path count (DWPC) metric. The left is an example graph subset showing select nodes and edges surrounding the Q92769 protein. To represent this protein, features are computed that measure the prevalence of a specific metapath between Q92769 protein and other proteins. The right demonstrates the process of calculating and weighting path counts using the DWPC metric. First, two features (pkp and ppp) can be used to represent the Q92769 protein, and for the specific metagraph, all paths are extracted from the network. Next, each path is associated with a path-degree product (PDP) measuring its specificity, which requires a damping exponent w; here, we set w=0.4. Finally, the PDPs are summed to obtain the DWPC.

An example of the computation process for the DWPC metric is shown in Figure 4.

### Machine Learning

Protein representations were used to train the model through machine learning. First, disease labels for proteins were obtained from the UniProt and OMIM databases. We collected the corresponding genes for schizophrenia from the OMIM database, and then mapped the genes to their protein products according to the UniProt database to tag these proteins. Next, we adopted four machine learning models as classifiers, random forest (RF), extreme gradient boosting (XGB), light gradient boosting machine (LGBM), and logistic regression (LR), which aimed to identify the schizophrenia-associated proteins based on the extracted metagraphs. For experimental settings, considering the sparsity of the protein-disease network where the number of unconfirmed protein-disease associations is much greater than the number of confirmed associations, we randomly sampled the nonschizophrenia (negative) proteins and schizophrenia (positive) proteins with ratios r∈{1,3,5,10,20} as the data set, and 10-fold cross-validation was used in model training and testing. Specifically, to avoid the bias of data splitting, we repeated the cross-validations 10 times in different random seeds and report the mean (SD) of four performance metrics: precision, recall, F1-score, and the area under the receiver operating characteristic curve (AUC).

To demonstrate the validity and superiority of the PPIK-based metagraph representations method, we compared our proposed work to other baseline methods developed for link prediction in networks, especially in the task of gene-disease association prediction. We selected three representative indices for performance comparison. The random walk with restart (RWR) index [23] is a random-walk algorithm based on similarity. RWR considers a random walker starting from node *x*, who will iteratively move to a random neighbor with probability *c* and return to node *x* with probability 1–*c*, and this process is repeated until it converges to a steady state. The average commute time (ACT) index [24] is also a random walk–based similarity index, which assumes that two nodes are considered to be more similar if they have a smaller average commute time. ACT is denoted by the average number of steps required by a random walker starting from node *x* to reach node *y* and from node *y* to node *x*. The Katz algorithm [25] is a path-dependent index that obtains global knowledge of the network topology; it can distinguish the impact of different neighbors by assigning them different weights.

### Ethical Considerations

UniProt is a protein database with the most complete sequencing and extensive annotation data available internationally. The STRING database contains data on various types of interactions, including direct physical interactions between proteins, indirect functional correlations, results extracted from PubMed abstracts, and outcomes predicted using bioinformatics techniques. The original data collection in the two databases received institutional review board (IRB) approval [26,27] and the scientific data in these two databases are freely available for users under a Creative Commons BY 4.0 license [28,29]. In addition, this study involved a secondary analysis, and the publicly available data sets were prepared with the intent of making them available for the public. The data available to the public are not individually identifiable and therefore the analysis would not involve human subjects. The Ethics Review Committee of Peking Union Hospital, Chinese Academy of Medical Sciences recognizes that the analysis of deidentified, publicly available data does not constitute human subjects research and therefore does not require IRB review; only biomedical research involving humans requires the approval of an ethics committee [30]. Several studies have used these two databases [31,32]. Over dozens of years, the UniProt and STRING databases have accumulated vast amounts of protein information and ethical approval was granted for collection of the original data with informed consent from the included patients. Analyses of these data have led to improvements in our ability to understand the molecular mechanisms of diseases, thereby promoting diagnosis and treatment. Since these data are open source, they remain publicly available for anyone in the research community to use.

## Results

### Model Evaluation and Comparison

The performance comparison results of the four machine learning models is shown in Table 2, with each metric representing the average score of the data set with different sampling ratios. The results showed that our proposed method could achieve relatively good performance with an AUC between 0.72 and 0.76. Specifically, with a ratio of 1, the RF model outperformed other models, demonstrating good discriminatory power with precision of 0.76, recall of 0.73, F1-score of 0.72, and AUC of 0.76. Therefore, we selected RF as the algorithm of choice for further investigation.

**Table 2:** Performance comparison of four machine learning models with different sampling ratios.

Model		AUC^a^, mean (SD)		F1, mean (SD)		Accuracy, mean (SD)		Recall, mean (SD)		Precision, mean (SD)
**Ratio=1**										
	LGBM^b^	0.75 (0.12)	0.7 (0.14)		0.72 (0.08)		0.71 (0.19)		0.73 (0.18)	
	LR^c^	0.75 (0.1)	0.73 (0.13)		0.73 (0.1)		0.76 (0.16)		0.72 (0.15)	
	RF^d^	0.76 (0.11)	0.72 (0.09)		0.72 (0.1)		0.73 (0.11)		0.76 (0.2)	
	XGB^e^	0.75 (0.11)	0.71 (0.13)		0.71 (0.1)		0.73 (0.13)		0.7 (0.16)	
**Ratio=3**										
	LGBM	0.73 (0.07)	0.52 (0.08)		0.72 (0.07)		0.62 (0.16)		0.47 (0.1)	
	LR	0.74 (0.07)	0.54 (0.06)		0.7 (0.09)		0.72 (0.11)		0.46 (0.11)	
	RF	0.75 (0.08)	0.5 (0.08)		0.69 (0.08)		0.64 (0.14)		0.44 (0.11)	
	XGB	0.76 (0.08)	0.53 (0.11)		0.71 (0.12)		0.65 (0.19)		0.48 (0.12)	
**Ratio=5**										
	LGBM	0.74 (0.09)	0.42 (0.11)		0.7 (0.1)		0.66 (0.18)		0.35 (0.14)	
	LR	0.73 (0.09)	0.43 (0.11)		0.68 (0.13)		0.68 (0.13)		0.34 (0.15)	
	RF	0.74 (0.07)	0.43 (0.11)		0.71 (0.14)		0.65 (0.15)		0.35 (0.13)	
	XGB	0.73 (0.08)	0.41 (0.1)		0.62 (0.1)		0.8 (0.12)		0.29 (0.09)	
**Ratio=10**										
	LGBM	0.75 (0.09)	0.32 (0.09)		0.74 (0.13)		0.64 (0.2)		0.23 (0.07)	
	LR	0.73 (0.1)	0.29 (0.08)		0.68 (0.13)		0.66 (0.11)		0.19 (0.07)	
	RF	0.75 (0.08)	0.29 (0.07)		0.69 (0.12)		0.69 (0.13)		0.19 (0.05)	
	XGB	0.75 (0.07)	0.32 (0.06)		0.72 (0.12)		0.66 (0.13)		0.22 (0.08)	
**Ratio=20**										
	LGBM	0.72 (0.07)	0.18 (0.07)		0.68 (0.13)		0.67 (0.13)		0.11 (0.05)	
	LR	0.73 (0.06)	0.2 (0.06)		0.72 (0.14)		0.65 (0.2)		0.13 (0.06)	
	RF	0.72 (0.06)	0.18 (0.04)		0.72 (0.09)		0.63 (0.12)		0.11 (0.03)	
	XGB	0.74 (0.06)	0.17 (0.03)		0.67 (0.13)		0.69 (0.15)		0.1 (0.02)	

^a^AUC: area under the receiver operating characteristic curve.

^b^LGBM: light gradient boosting machine.

^c^LR: logistic regression.

^d^RF: random forest.

^e^XGB: extreme gradient boosting.

### Comparison to Baseline Methods

We compared the AUC values of the three baseline methods (RWR, ACT, and Katz index) on the PPI and PPIK networks, respectively. The performance of these methods on the PPIK network was better than that on the PPI network under most circumstances, according to the AUC values, as shown in Table 3. This demonstrated that protein keywords can indeed complement and enrich the PPI network.

We further compared AUC values between the baseline methods and our proposed method on the PPIK network. Our method achieved better performance on the whole, which outperformed the Katz, ACT, and RWR methods by 0.156, 0.294, and 0.457, respectively, as shown in Table 3. Thus, it can be concluded that metagraph representation is a feasible method for network topological features.

We found that the AUC values of these baseline methods were quite low overall, which may be due to the difficulty for these methods to accurately capture the characteristics of the heterogeneous and sparse network we constructed. However, our framework can effectively capture various features in the network and demonstrated good performance.

**Table 3:** Comparison of performance (area under the receiver operating characteristic curve values) of three baseline models and our proposed metagraph method on protein-protein interaction (PPI) and PPI-keyword (PPIK) networks.

Network	RWR^a^	ACT^b^	Katz	Metagraph
PPI	0.521	0.333	0.269	N/A^c^
PPIK	0.604	0.466	0.303	0.76

^e^RWR: random walk with restart.

^b^ACT: average commute time.

^c^N/A: not applicable.

### Potential Schizophrenia Risk Genes Prioritization

We focused on the results from RF, as the model with the best performance, for the discovery of potential schizophrenia-causing proteins. As the goal of our study was to identify new proteins associated with schizophrenia, we excluded confirmed protein-schizophrenia associations in the database and retained the top 20 proteins identified as risk proteins for schizophrenia. We then transformed our predicted disease proteins to their producer gene IDs based on UniProt. The top 20 predicted genes and their probability scores are listed in Table 4.

**Table 4:** Top 20 schizophrenia-associated risk genes predicted from the random forest model.

Uniprot ID	Gene symbol	Predicted probability
9606.ENSP00000362978	*EYA3*	0.94305
9606.ENSP00000380602	*CNTN4*	0.92609
9606.ENSP00000432083	*HSPA8*	0.871386
9606.ENSP00000298910	*LRRK2*	0.865878
9606.ENSP00000379138	*AFP*	0.862006
9606.ENSP00000344456	*CTNNB1*	0.845441
9606.ENSP00000369050	*CYP1A1*	0.809024
9606.ENSP00000393312	*FGFR1*	0.775439
9606.ENSP00000265563	*PRKAR2A*	0.772953
9606.ENSP00000347184	*HTT*	0.770366
9606.ENSP00000266058	*SLIT1*	0.75607
9606.ENSP00000308546	*MEPCE*	0.744564
9606.ENSP00000273853	*CENPC*	0.738029
9606.ENSP00000423673	*NDUFA13*	0.73425
9606.ENSP00000345502	*PDE4D*	0.731391
9606.ENSP00000301015	*PIEZO1*	0.721304
9606.ENSP00000263246	*PACSIN2*	0.715347
9606.ENSP00000398131	*GSPT1*	0.709701
9606.ENSP00000373783	*LOXL2*	0.70657
9606.ENSP00000352264	*CD2AP*	0.69778

## Discussion

### Principal Findings

In this work, we proposed an optimized model to enhance schizophrenia-related genes prediction. Our framework identified 20 potential schizophrenia risk genes: *EYA3, CNTN4, HSPA8, LRRK2, AFP, CTNNB1, CYP1A1, FGFR1, PRKAR2A, HTT, SLIT1, MEPCE, CENPC, NDUFA13, PDE4D, PIEZO1, PACSIN2, GSPT1, LOXL2*, and *CD2AP*. Given these results, it is promising to examine what our framework produced. Toward this end, we here further evaluate the top risk genes associated with schizophrenia based on publications to support our predictions and analyze these genes from four different perspectives: function, gene-set enrichment, signaling pathways, and gene character (ie, verification). The evidence for these predictions can be found in the up-to-date literature, which has proven to be reasonable.

The model adopted in our study is a metagraph representation based on the PPIK network framework. We used this model to identify potential schizophrenia-associated genes. This model attempted to integrate the PPI network with the keywords that describe the biological aspects of the proteins to ultimately form a PPIK network for disease gene prediction. The heterogeneous network contains rich information, involving not only protein interactions with one another but also their functional and structural similarities, which could better compensate for the limitations of the standard PPI network. Based on the PPIK network, we used metagraphs to solve the disease protein classification problem. This type of graph structure can thoroughly capture the topology of a heterogeneous network. By permuting all instances of tiny motifs, we can obtain all biological arrangements between proteins and keywords on the PPIK network, allowing an accurate representation of each protein using a series of metagraphs. In particular, the DWPC indicator was used to quantify metagraph representations, which could capture the prominent feature of metagraphs. Finally, we built a classifier using four advanced machine learning algorithms for disease proteins based on these metagraph representations and selected the best-performing model for disease gene prediction. The utilization of machine learning models to prioritize selected proteins according to predicted probabilities enhanced the interpretability of the results. Our proposed framework can be a practical and efficient prediction model. Of the four machine learning methods, RF achieved the best overall performance with precision of 0.76, recall of 0.73, F1-score of 0.72, and AUC of 0.76. Therefore, the framework using the RF classifier demonstrates superior predictive power.

### Analysis Based on Publications

#### Overall Approach

**Figure 5:**
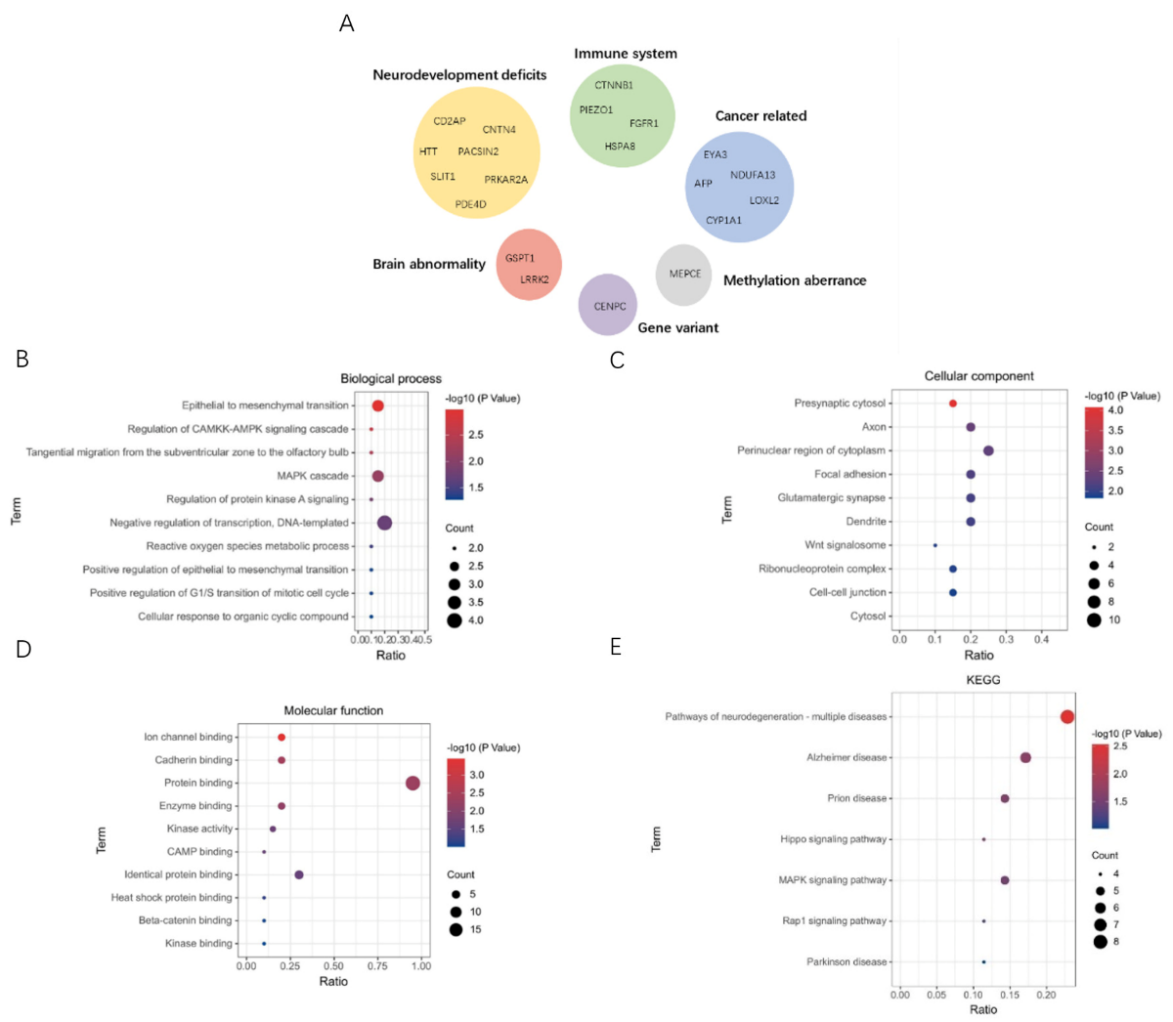
Analysis of the top 20 potential schizophrenia risk genes from different perspectives. (A) Results of gene function clustering analysis, with each cluster containing a group of genes with similar function. (B-D) Result of gene-set enrichment analysis on biological process, cellular component, and molecular function terms, respectively, representing the cluster of gene products at three levels based on biological properties. (E) Result of signaling pathways enrichment analysis, with each signaling pathway made up of a set of genes that share common traits and mechanisms.

We searched for evidence from the literature to further validate our method. We evaluated the two new genes with the highest prediction scores and interpreted the potential associations between these genes and diseases from three perspectives, including gene-set enrichment analysis, signaling pathways enrichment analysis, and gene function clustering analysis, as shown in Figure 5.

#### Gene Functional Clustering Analysis

By dividing gene sets according to relevant function, six categories were mainly formed, including neurodevelopment, immune system, brain abnormality, cancer, gene variant, and methylation, as shown in Figure 5A.

Genes related to neural development defects play a role in causing functional disorders in the synapses. The neural development disorders caused by disruptions in synaptic communication can lead to schizophrenia. It is suggested that these genes mediate glutamatergic signal transduction by partially encoding glutamate receptors, which are important for regulating the maturation and function of neurons [33].

The immune system–related genes are a group of genes involved in immune pathways. Immune dysregulation is closely associated with schizophrenia [34]. The role of inflammatory mechanisms in schizophrenia is supported by the impact of immune dysregulation and alterations in neuroinflammatory pathways in schizophrenia. Neurotransmitter dysfunctions resulting from cytokine-induced neuroinflammation through microglial activation lead to the inflammatory process and neurodegeneration in schizophrenia. The nuclear factor-κB signaling pathway plays a role in immune response regulation, synaptic plasticity, and memory, which is also associated with schizophrenia.

Abnormal expression of genes in the brain seems to be specifically active in brain neurons. Patients with schizophrenia often exhibit low levels of cognitive functioning, with decreased connectivity of whole-brain function detected in computed tomography scans and low frontal lobe function of the brain detected in positron emission tomography scans. These findings are considered to be predicators of structural abnormalities in the brain [35].

In cancer-related genes, a GWAS revealed a strong correlation between cancer and schizophrenia risk genes, which is further supported by a bidirectional epidemiological correlation, common genetic changes, and biological mechanisms between the two phenotypes [36].

Gene variants caused by gene mutation increase the susceptibility to disease. Genome-wide enrichment of rare deletions and duplications and a higher rate of de novo copy number variations have been reported in schizophrenia cases. For example, gene mutations cause truncation variants in proteins, resulting in the absence of crucial structural domains or functional regions. Additionally, rare structural variants can disrupt genes essential for neural development pathways, specifically those responsible for synapse formation and function, and polymorphic sites of single nucleotide polymorphisms (SNPs) can interact with each other, thereby increasing the likelihood of schizophrenia.

In genes with abnormal methylation, DNA methylation at CpG sites can regulate gene expression during disease processes [37]. Moreover, methylation can control the onset and progression of the neurodegeneration associated with different cell signaling pathways and dynamically regulate differentiated neurons. This process suggests that there is a correlation between neurodegenerative diseases with implications for schizophrenia [38].

#### Gene-Set Enrichment Analysis

Figure 5B demonstrates the results of gene-set enrichment analysis on biological processes. Among these biological processes, regulation of the calcium/calmodulin-dependent protein kinase kinase (CAMKK)–AMP-activated protein kinase (AMPK) signaling cascade was among the significantly correlated pathways, including the LRRK2 and HTT genes, with a prediction probability of 0.87 and 0.77 in the RF classifier, respectively. Impaired cognitive function is an important feature of patients with schizophrenia. This has been linked to increased levels of free radicals and decreased levels of antioxidants, which can activate a range of inflammatory factors, ultimately causing damage to brain cells. In the nervous system, excessive autophagy causes cell death, which in turn leads to damage to cognitive function. Inflammatory stimuli can also cause neurotoxicity and contribute to cognitive dysfunction. AMPK is a crucial kinase in regulating metabolism, which participates in a variety of basic biological processes upon activation, such as cell growth, proliferation, apoptosis, and autophagy. The upstream kinase CAMKK2 can activate the AMPK mechanism when triggered by intracellular calcium ions. By activating AMPK, oxidative stress [39], cellular autophagy, and neuroinflammatory processes can be modulated, all of which are closely related to neurodegenerative diseases, thus providing insight into schizophrenia [40].

Figure 5C demonstrates the results of gene-set enrichment analysis on cellular components. Of these cell components, the Wnt signalosome pathway had a significant correlation, involving the LRRK2 and CTNNB1 genes, and the prediction probability in the RF classifier of the two genes was 0.87 and 0.85, respectively. The Wnt/β-catenin signaling pathway plays a crucial role in the central neurodevelopment of the brain and helps maintain its proper function. Multiple schizophrenia susceptibility genes have been identified in this pathway. One such gene is *GSK3*, a key regulator of signal transfer, and changes to its expression level may be a contributing factor to schizophrenia. Additionally, this pathway plays an important role in the development of dopaminergic neurons. Furthermore, synaptogenesis, axoplasmic transport, and abnormalities in learning and memory functions in the pathway may be associated with certain symptoms of schizophrenia [41].

Figure 5D demonstrates the results of gene-set enrichment analysis on molecular functions. Among these molecular functions, the heat shock protein (HSP) binding pathway showed a significant correlation. This process involves the HSPA8 and HTT genes with a prediction probability in the RF classifier of 0.87 and 0.77, respectively. HSPs are a group of protein chaperones that protect cells from stress and play a vital role in neurodevelopment. In particular, HSP60 has been found to have dual pro- and anti-inflammatory effects, which have implications for the development of neuropsychiatric disorders. The central nervous system of patients with schizophrenia undergoes autoimmune-mediated processes, which increase the antibody responses to HSP70 and HSP90AB1 proteins. This indicates a link between HSPs and the onset of schizophrenia [42].

#### Signaling Pathways Enrichment Analysis

Figure 5E presents the results of signaling pathways enrichment analysis. Of various signaling pathways, the most significantly correlated pathway was the Rap1 signaling pathway, involving the CTNNB1 gene with a prediction probability of 0.85 and the FGFR1 gene with a prediction probability of 0.78 in the RF classifier. The Rap1 signaling pathway has been shown to be involved in synaptic plasticity, excitation, learning, and memory by inhibiting the release of L-type calcium channel–dependent neurotransmitters, suggesting a link with schizophrenia [43].

#### Verification of the Top Two Genes

We further examined the role of the top two genes among the top 20 predicted genes. The protein encoded by the EYA3 gene had a prediction probability of 0.94 in the RF classifier, which acts as a transcriptional activator and plays a role in the growth and development process, suggesting a link with human behavior or neural development. This gene may also induce the occurrence, progression, and metastasis of tumors. Studies have found that some genes highly expressed in cancer cells are closely tied to synaptic plasticity. These genes are often expressed at high levels in the mature neurons of the hippocampus and endbrain and can regulate dopamine dysfunction caused by dopamine D2 receptor expression. These genes affect the nervous system and regulate memory and emotions, indicating a strong correlation with schizophrenia [44]. Therefore, we speculated that *EYA3* may be a potential risk gene for schizophrenia.

Neuronal cell adhesion molecules (CAMs) allow for interactions between nerve cells, thus providing support for development of nervous system. Contactins (CNTNs) showed a prediction probability of 0.93 in the RF classifier. As a special subclass of immunoglobulin CAMs, CNTNs have been found to play a critical role in the functioning of neurons and glial networks. As schizophrenia is believed to have its origins in neural development [45], *CNTN4* may be an important candidate gene for schizophrenia. Additionally, a nonsynonymous SNP of this gene was associated with the effects of olanzapine and risperidone on negative symptoms of schizophrenia, indicating that *CNTN4* may serve as a risk signal for the identification of schizophrenia.

### Feature Analysis

**Figure 6:**
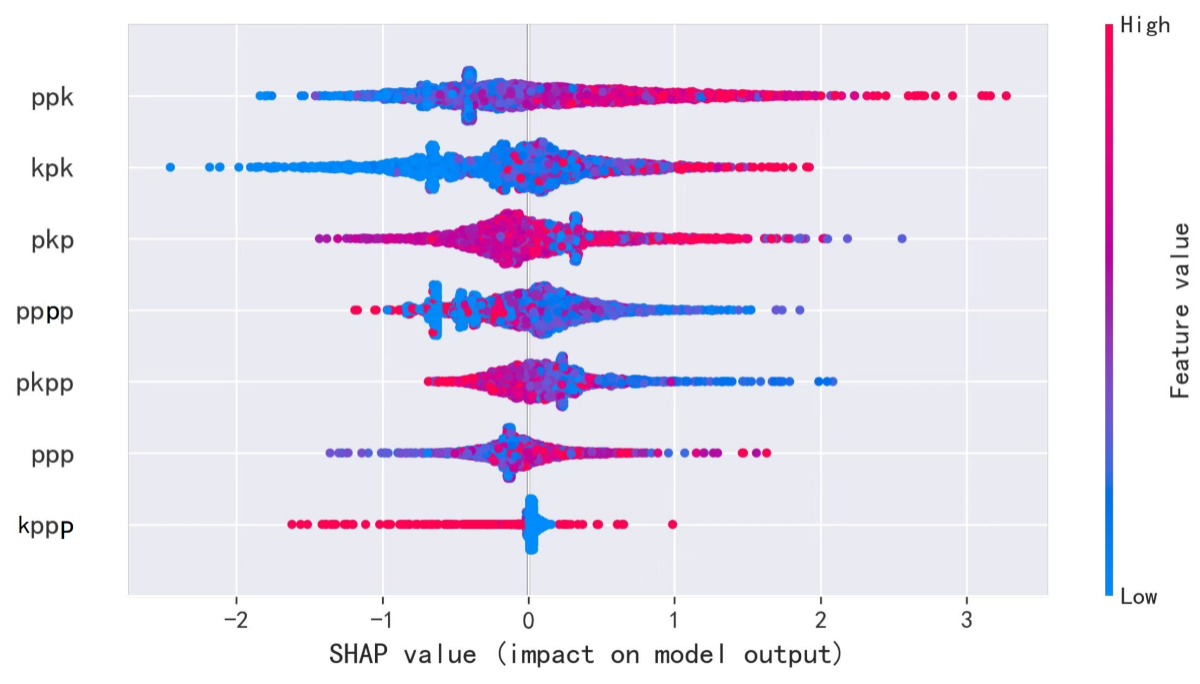
Feature explanation of RF model using the Shapley additive explanation (SHAP) value. The importance of each feature decreases from top to bottom, and among these metagraph features, the "ppk," "kpk," "pkp," and "ppp’" features are positively correlated with schizophrenia; the features "pppp" and "pkpp" are negatively correlated; and "kppp" has little influence on the model.

We used the Shapley additive explanation value to analyze the importance of the metagraph features contributing to our model. The features are arranged in descending order of importance in Figure 6. We found that features containing keywords ranked on top, indicating their important role for the prediction capability of the model and their ability to capture more information and provide a better representation of each protein. Among them, larger values of the features *pk*, *kpk*, *pkp*, and *pp* indicate a higher likelihood of being a schizophrenia-associated protein, which indicated that these metagraph features reflected more characteristics describing the molecular mechanism related to the pathogenesis of schizophrenia, whereas other metagraph features seemed to have the opposite impact. The interactions between proteins form the basis of many crucial life activities, and they are of great significance in unraveling complex biological processes in living organisms and understanding the molecular mechanisms of diseases. In particular, the protein complexes that are tightly bound and have specific biological functions are closely related to diseases. The *ppk*, *kpk*, *pkp*, and *ppp* metagraphs are denoted as tightly coupled proteins with specific functions. For example, the 14-3-3 epsilon protein and the reticulon-4 receptor protein are associated with the same biological process of cerebral cortex development. Therefore, such metagraphs may be positively correlated with the incidence of diseases. By contrast, the *pppp*, *pkpp*, and *kppp* metagraphs each contained four nodes. An increase in the number of nodes in the pathway may result in some redundancy of features and thus weaken their meaning. Such paths typically include the binding and interaction of two protein complexes that are not closely linked. For example, the cadherin-related family member 1 protein and the meurexin-2 and neurite extension and migration factor protein complex are associated with the same biological process of replication factor C subunit 2, which therefore have a diminished impact on diseases or may even be negatively correlated with diseases. However, all of these features offer important information for the description of a network, showing various aspects of the network characteristics, and thus these features should be fully employed to represent the proteins.

### Primary Contribution

In our study, we chose the classifier with the best performance for disease gene prediction. The proposed framework successfully discovered associations hidden from the network and achieved relatively favorable results. The main benefits of our framework are as follows. First, the keywords we considered in the network described rich biological properties of proteins and thus encompassed reasonable predictive power for disease genes. Second, the vector representations for proteins we constructed improved our ability to capture the topological arrangement on the PPIK network for interactions between both proteins and keywords, and each protein can provide a comprehensive representation of its characteristics from various aspects. In addition, it is noteworthy that this framework has general applicability, which can not only be applied to schizophrenia but can also be fine-tuned to achieve better performance by tailoring to other specific diseases.

### Limitations

Despite these benefits, our method still has some limitations. Notably, our method failed to adequately leverage pleiotropy and diverse biological features. To resolve this issue, we can propose a strategy to build a heterogeneous network covering multimodal data. Based on this comprehensive network, we can excavate the potential characteristics of the biological entities and relationships among them to explore candidate disease genes. In addition, our method was based on machine learning techniques, and effective algorithms are required to transform features into a training data set. However, these algorithms are complex and cannot fully reflect the biological information of various aspects in the network. For future work, we can employ deep learning approaches such as convolutional neural networks and autoencoders into our framework for network embedding, which can capture both the structural and nonstructural properties of networks as well as dig out implicit features, thus leading to better predictive performance.

### Conclusions

Predicting potential associations between diseases and genes is crucial to the diagnosis and treatment of many diseases. In this work, we proposed an optimized method to mine and predict schizophrenia-associated risk genes. We incorporated PPI and biological keywords of proteins to construct a PPIK network. We then extracted features from the PPIK network and proposed metagraph representations for proteins. Further, we trained and optimized our model using four machine learning algorithms, including RF, XGB, LGBM, and LR, for disease protein prediction. Our method achieved relatively good performance, outperforming the RWR, ACT, and Katz baseline methods. We applied RF, as the machine learning model with better comprehensive performance, to make the prediction. In particular, we mapped these proteins to their gene IDs and obtained the top 20 novel potential schizophrenia-associated genes. Finally, we performed feature analysis and searched for evidence in the literature to explain the potential association between specific genes and diseases. The results indicate that application of our approach was quite reasonable and reliable in the discovery of schizophrenia-associated risk genes. Overall, our method provides a means to enrich protein interactions with more detailed information, which can encourage the deeper mining of relations between proteins. This method is likely to contribute to research on schizophrenia pathology and can offer some guidance for the diagnosis and treatment of schizophrenia. In addition, this approach can help to save time and costs, thereby facilitating basic research. After more testing and optimization in independent samples, these results are expected to be applied in future clinical practice and clinical trials.

### Data Availability

The data sets generated and analyzed in this study are available from the corresponding author on reasonable request.

## Abbreviations

ACT: average commute time
AMPK: AMP-activated protein kinase
AUC: area under the receiver operating characteristic curve
CAM: cell adhesion molecule
CAMKK: calcium/calmodulin-dependent protein kinase kinase
CNTN: contactin
DWPC: degree-weighted path count
GCN: graph convolutional network
GRAIL: Gene Relationships Among Implicated Loci
GWAS: genome-wide association study
HSP: heat shock protein
IRB: institutional review board
LGBM: light gradient boosting machine
LR: logistic regression
MAGENTA: meta-analysis gene-set enrichment of variant associations
PDP: path-degree product
PPI: protein-protein interaction
PPIK: protein-protein interaction-keyword
RF: random forest
RWR: random walk with restart
SNP: single nucleotide polymorphism
XGB: extreme gradient boosting
